# Effect of Emi1 gene silencing on the proliferation and invasion of human breast cancer cells

**DOI:** 10.1186/s12860-023-00494-1

**Published:** 2023-12-01

**Authors:** Ying Kuang, Shengwen Huang, Shifan Tang, Zhaozhen Zhuo, Keyan Linghu

**Affiliations:** Department of Antenatal Diagnostic Centre, Guizhou Provincial People’s Hospital, No. 56 East Zhongshan Road, Guizhou, 550000 Guizhou China

**Keywords:** Emi1 gene, Breast cancer, Proliferation, Invasion

## Abstract

Breast cancer is the most common malignant tumour in women. The early silk-splitting inhibitor protein 1 Emi1 is responsible for mediating ubiquitin protein degradation. The present study investigated the effects of the decreased expression of the Emil gene on the proliferation and invasion of breast cancer cells. The interference efficiency of small interfering ribonucleic acid (siRNA) was quantitatively verified using fluorescence real-time quantitative polymerase chain reaction (RT-qPCR) and Western blotting, and the effect of Emi1 gene silencing on cell vitality and invasion was determined using MTT and Transwell assays, respectively. The expression of the proliferation genes programmed cell death receptor 4 (PDCD-4), fatty acid synthase ligand (FasL), PTEN and RhoB, along with the invasive genes Maspin, TIMP3 and RECK, was measured using fluorescence RT-qPCR. In breast cancer cells, siRNA successfully reduced the expression of the Emi1 gene, and the expression level of the cell proliferation genes PDCD-4, FasL, PTEN and RhoB, along with invasive genes Maspin, TIMP3 and RECK, decreased significantly (P < 0.05). Furthermore, Emi1 gene silencing reduced the proliferation and invasion abilities of MDA-MB-231 and SUM149PT cells by reducing the expression of proliferative and invasive genes.

**Supplementary Information:**

The online version contains supplementary material available at 10.1186/s12860-023-00494-1.

## Introduction

Breast cancer is the most common malignant tumour in women and the main cause of cancer deaths in women. The incidence of breast cancer has been reported to account for 7–10% of all malignant tumours. Reports show that globally, approx. 1.2 million women suffer from breast cancer every year, and 500,000 die from the disease [1]. In Europe and North America, the incidence of breast cancer accounts for the highest proportion of malignant tumours in women. The growth rate of breast cancer incidence in China is almost the same as the growth rate of global breast cancer incidence. It is expected that by 2021, the incidence of breast cancer in China will rise to > 100 cases per 100,000 women, or > 2.5 million cases in total [2]. Traditional therapies, such as surgery, chemotherapy and radiotherapy, essentially cause adverse effects in patients. With the in-depth study of the molecular mechanisms of tumour cell growth, proliferation and apoptosis, modern tumour therapy has entered the era of biological therapy, and targeted tumour therapy has become possible.

The early fission inhibitory protein 1, Emi1, which belongs to the F-box protein family (also known as FBOX5), can be involved in the formation of the Skp1-Cull-F-box-protein ubiquitin connection enzyme and mediates ubiquitin protein degradation [3, 4]. The expression of Emi1 in colon cancer has been reported as increased and associated with the malignancy and poor prognosis of tumours. In order to further study the mechanism of the Emi1 gene in the development of breast cancer, small interfering ribonucleic acid (siRNA) was infected with human breast cancer cell lines. After screening and verification, a stable reduction of an Emi1 gene breast cancer cell model was successfully established.

Studies have reported that the programmed cell death receptor 4 (PDCD-4), fatty acid synthase ligand (FasL), PTEN and RhoB genes are related to the proliferative regulation of breast cancer cells and that Maspin, TIMP3 and RECK genes are related to the invasion regulation of breast cancer cells [5–7]. The present study showed that Emi1 gene silencing can reduce the biological activity of MDA-MB-231 and SUM149PT cells by decreasing the expression of proliferative and invasive genes. After finding that the knockdown (KD) of Emi1 reduced the proliferation and invasion abilities of breast cancer cells, the authors further explored the molecular mechanism by which Emi1 affects breast cancer cells.

## Materials and methods

### Experimental materials

The human breast cancer cell strains MDA-MB-231 and SUM149PT were purchased from the Shanghai Institute of Cell Biology at the Chinese Academy of Sciences, and lentiviral scrambling vector LV-Emi1-ribonucleic interference (RNAi) and negative-control viral vector LV-Emi1-RNAi were obtained from Shanghai Genechem. Reverse transcription (RT) primers were obtained from GeneRay (CA, USA), and microRNA polymerase chain reaction (PCR) was obtained from Thermo Scientific (MA, USA). Both M-MLV kits and deoxynucleotide triphosphates were obtained from Promega (MA, USA), and MTS cell viability detection kits were obtained from Shanghai Sangon. Lipofectamine™ 2000 was obtained from Thermo Scientific (MA, USA), and TRIzol™ was obtained from Shanghai Pufei. Finally, TaqMan PCR (qPCR) primers were obtained from Shanghai Genechem, and TaqMan PCR kits were obtained from Thermo Scientific (12,574,035, MA, USA).

### Experimental methods

#### Total RNA extraction

The cell lines MDA-MB-231 and SUM149PT were purchased from the Cell Resource Center, Shanghai Academy of Life Sciences and cultured in Dulbecco’s modified Eagle medium containing 10% foetal serum. After the cell density reached approx. 70–80%, the experimental (KD) group was transfected with a lentiviral vector (LV-Emi1-RNAi) containing an effective interference sequence against Emil. The negative control (NC) group was transfected with Emi1 without effective infection, and the empty control (CON) group was transfected with a chronic viral vector, LV-CON. The cells were subsequently collected. The total RNA was extracted with TRIzol lysate, and the concentration and quality of the RNA were measured using a NanoDrop™ 2000/2000 C spectrophotometer.

#### Reverse transcription to obtain cDNA

For the microRNA reverse transcription, the primer information was as follows: for the endogenous reference gene GAPDH, the upstream primer sequence was TGACTTCAACAGCGACACCCA, and the downstream primer sequence was CACCCTGTTGCTGTAGCCAAA. The amplified fragment was 121 bp. For the target gene Emi1, the upstream primer sequence was CGAGAAGGCTGTGGATTTGAT, and the downstream primer sequence was ACCAGGCAGGGGACCTATTTT. The amplified fragment was 117 bp. After the primers were prepared, RNA RT and two-step real-time RT-PCR were performed, and a dissolution curve was created.

#### Design of ribonucleic acid targeting the Emi1 gene

The RNAi lentiviral vector was prepared via synthesis, construction, identification, sequencing and extraction. The siRNA lentiviral expression vector targeting the Emi1 gene included three target sequences as follows: Emi1-RNAi (1): TCGCTGTAATTCACCTGCAAA; Emi1-RNAi (2): CCAGACCAATATCCCAACAAA; and Emi1-RNAi (3): CGGTGTAGTATCCTGAGGTTT. The tool carrier number was GV248, the frame structure was hU6-MCS-Ubiquitin-EGFP-IRES-puromycin, the control number was CON077, and the control insert sequence was TTCTCCGAACGTGTCACGT.

#### RNAi lentivirus packaging, concentration and purification, titer determination and Infection of target cells

The amount of virus used was calculated. Human breast cancer cells MDA-MB-231 and SUM149PT were inoculated on 12-well plates in accordance with the KD group (LV-Emi1-RNAi, named according to the lentiviral vector containing the effective interference sequence target, Emil), the NC group (without effective Emi1 infection) and the CON group (the chronic viral vector, LV-CON). The cells were kept in an incubator containing carbon dioxide. Lentiviral infection was performed after three days, and the medium was changed after 12 h. The infection efficiency of the virus was observed under a fluorescence microscope. When the fluorescence rate was approx. 70–80% and the confluence rate was approx. 80%, the cells were collected. Next, RNA was extracted 4 days after cell proliferation and infection in each group, and the level of Emi1 mRNA expression in each group was determined.

#### Real-time quantitative polymerase chain reaction of Emi1 and possible targeted proliferation and invasion-related genes

The LV-Emi1-RNAi virus particles containing the target gene were used to infect the target cells. The fluorescence of the cells was observed using a fluorescence microscope; the infection rate was > 50%. The cells were collected, RNA was extracted and reverse transcribed into cDNA, and the expression of Emi1 mRNA in each group was determined. When detecting the mRNA content of Emi1, the cells were seeded in a 12-well cell culture plate. After 24 h of transfection, the medium was discarded. After washing twice with phosphate-buffered saline, TRIzol lysate was added, and the total RNA was extracted. The RT was conducted with 1-μg RNA, and cDNA was obtained and amplified according to the following procedures.

The reaction mixtures were incubated at 50 °C for 15 min, followed by 95 °C for 5 min; next, 40 PCR cycles were performed with the following temperature profiles: 95 °C for 15 s, 60 °C for 30 s and 72 °C for 1 min; the amplification curve and the number of take-off cycles (Ct) were obtained. The mRNA content was calculated according to 2 − ΔΔCt. TaqMan PCR kits were obtained from Thermo Scientific (12,574,035, the United States). The Platinum Taq enzyme was used. The primer sequence information is as follows: GAPDH: F: TGACTTCAACAGCGACACCCA; R: CACCCTGTTGCTGTAGCCAAA. Emi1: F: CGAGAAGGCTGTGGATTTGAT; R: ACCAGGCAGGGGACCTATTTT. PDCD-4: F: TTGAGCACGGAGATACGAAC; R: GTCCCGCAAAGGTCAGAAAG. FasL: F: GGCCTGTGTCTCCTTGTGAT; R: TGCCAGCTCCTTCTGAAGTA. PTEN: F: TGGATTCGACTTAGACTTGACCT; R: GGTGGGTTATGGTCTTCAAAAGG. RhoB: F: ATCCCCGAGAAGTGGGTCC; R: CGAGGTAGTCGTAGGCTTGGA. Maspin: F: GCCAGGAGCACGGATCCT; R: GTTGTGCCTGATGTAAATAAAGG. TIMP3: F: CAGGTCGCGTCTATGATGGC; R: AGGTGATACCGATAGTTCAGCC. RECK: F: AGTGCGGGTGCATTGTGTT; R: TTCACAGCAGCCTAAGCCAAC.

#### Western blotting for Emi1 protein level detection

The total protein was extracted from the infected cells; the protein concentration was determined using the bicinchoninic acid assay method, and the protein level of Emi1 was detected using Western blotting (WB), as previously described [7]. The primary antibody (anti-Emi1 antibody) (ab215765, Abcam, Cambridge, UK) was diluted at a ratio of 1:200 and incubated overnight at 4 °C; the secondary antibody (Anti-rabbit IgG, HRP-linked Antibody, #7074, Cell Signaling Technology, MA, USA) was diluted at a ratio of 1:2,500 ~ 3,000 and incubated at room temperature for 1 h. The protein bands were scanned by a computer, and the grey level was analysed by software. The expression rate of Emi1 (Emi1/GAPDH [ratio of grey value])% was determined using GAPDH as an endogenous reference.

#### Cell viability test

To detect cell viability, the cells were seeded in a 96-well culture plate. After 24 h of transfection, 20 μl of MTS detection solution was added to the culture system. After 4 h of continuous incubation, the culture plate was placed in a microplate reader, and the absorbance (OD) value at a wavelength of 490 nm was measured [8].

#### Cell invasion detection

To detect cell invasion, the cells were seeded in a Transwell chamber. After 24 h of transfection, the cell plate at the bottom of the chamber was removed and stained with DAPI staining solution. The numbers of cells in three high-power fields were counted under a fluorescence microscope [8].

### Statistical methods

The data were recorded and processed using the SPSS 20.0 software. First, the data were tested to see if they corresponded to a normal distribution; if they did, the data between the two groups were analysed by a two-tailed t-test. A P value of < 0.05 indicated that the difference was statistically significant. A nonparametric test was used for non-normal distribution.

## Results

### Expression and knockdown of the Emi1 gene in human breast cancer cell strains

In the present study, Emi1 KD was conducted to investigate the effect on breast cancer cells. The detection of the Emi1 gene expression in human MDA-MB-231 and SUM149PT cell strains was conducted using RT-qPCR. The results are shown in Table 1. The Emi1 gene was highly expressed in human MDA-MB-231 and SUM149PT cell lines.

**Table 1:** Expression of the FBXO5 gene in human MDA-MB-231 cell strains

Group	Well 1	Well 2	Well 3
FBXO5 Group	20.08	20.15	20.15
GAPDH Group	13.25	13.24	13.15
∆Ct	6.83	6.91	7

Note: The ∆Ct of the endogenous reference gene well is 0.100; the ∆Ct of the target gene well is 0.070

∆Ct = target gene Ct value-endogenous reference gene Ct value; when ∆Ct ≤ 12, the gene expression abundance in the cell is high

**Fig. 1:**
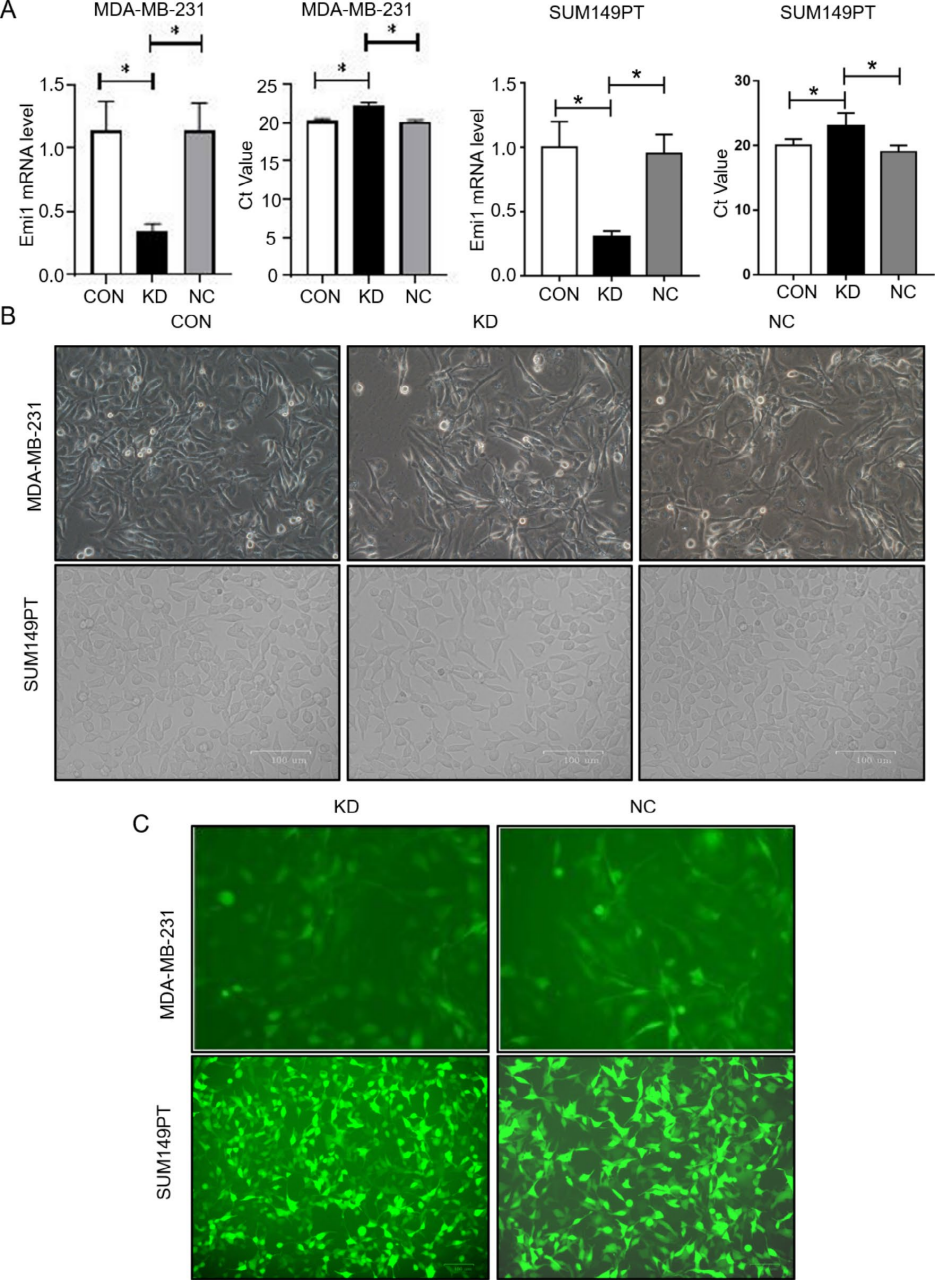
(**A**) The expression level of Emi1 of each group measured by RT-QPCR. (**B**) The bright field view of MDA-MB-231 and SUM149PT cells under a microscope after lentivirus infection. (**C**) Fluorescence images of MDA-MB-231 and SUM149PT cells under a microscope after lentivirus infection. CON: control cells transfected with LV-CON, KD: cells transfected with Emi1-RNAi, NC: cells transfected with LV-GFP virous. All data were expressed as Mean ± SD, *P < 0.05

In order to further explore the function of the Emi1 gene, a lentivirus containing Emi1-siRNA was constructed to decrease the expression level of Emi1 by infecting MDA-MB-231 and SUM149PT cells. In terms of Emi1 mRNA expression, the expression level was significantly lower in the KD group than in the CON group; however, there was no significant difference between the CON group and NC group (Fig. 1A). Green fluorescence (Fig. 1B and C) in the MDA-MB-231 cells was observed under an inverted fluorescence microscope. Counting and formula calculations revealed the transfection efficiency to be 90%. The results showed that Emi1-siRNA successfully decreased the expression of the Emi1 gene.

**Fig. 2:**
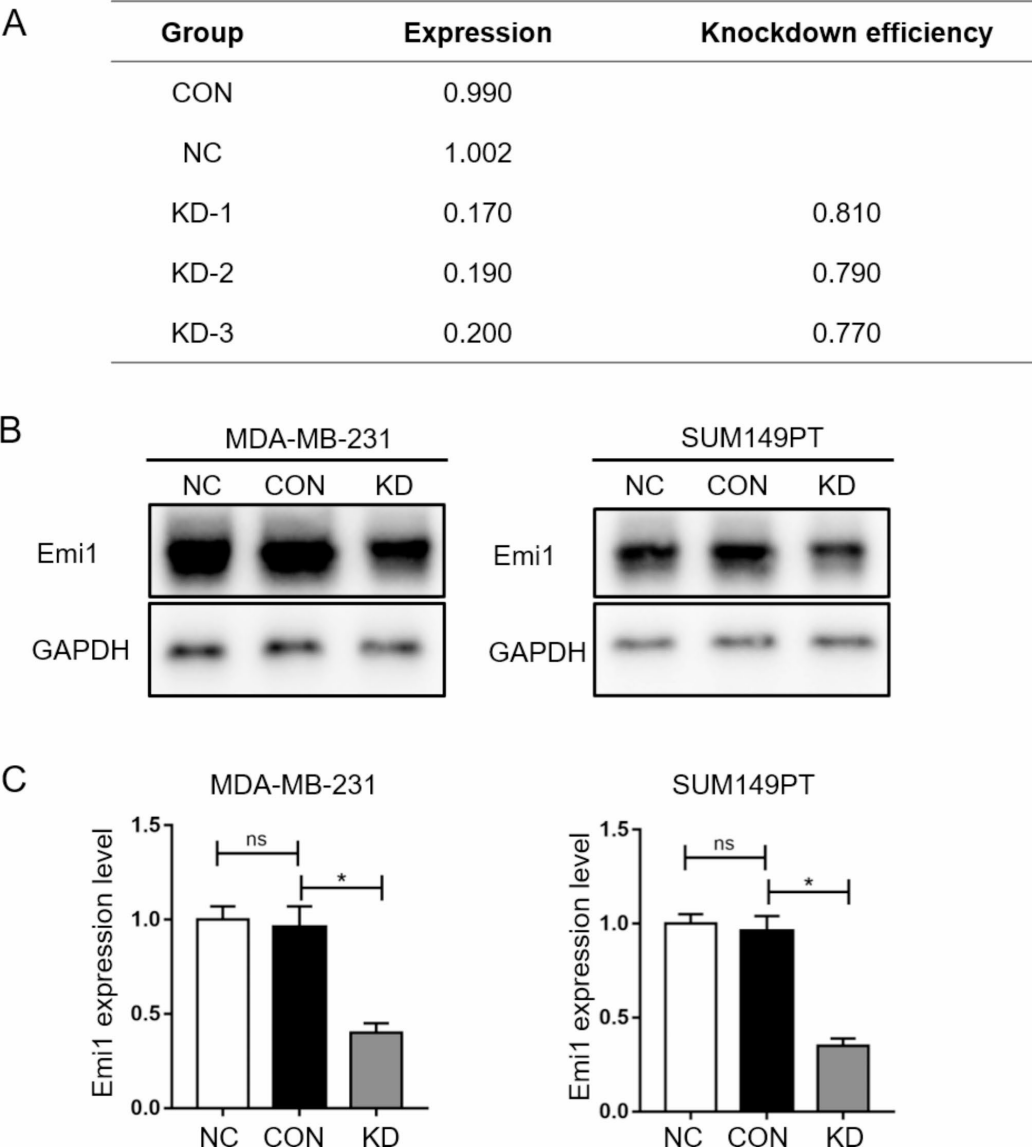
(**A**) The expression level of Emi1 of each group measured by RT-QPCR. (**B**) Immunoblotting results of Emi1 expression in different groups. (**C**) Statistic graph of Emi1 relative expression. CON: control cells transfected with LV-CON, KD: cells transfected with Emi1-RNAi, NC: cells transfected with LV-GFP virus. All data were expressed as Mean ± SD, ns means no significance, *P < 0.05

The expression of Emi1 in MDA-MB-231 and SUM149PT cells was detected using RT-qPCR and WB. The results are shown in Fig. 2. In the RT-qPCR detection of the Emi1 gene KD efficiency, siRNA successfully knocked down the Emi1 gene in both MDA-MB-231 and SUM149PT cells (KD efficiency of > 75%) (Fig. 2A). The WB results showed that Emi1-siRNA transfection could knock down Emi1 protein expression. The WB results also showed that the expression of the Emi1 protein was lower in the KD group than in the CON group and that the level of Emi1 in the KD group decreased (KD efficiency of > 50%) (Fig. 2B and C). The results revealed that Emi1-siRNA successfully decreased the expression of the Emi1 gene at the protein level.

### Knockdown of Emi1 reduced the migration ability of MDA-MB-231 cells

**Fig. 3:**
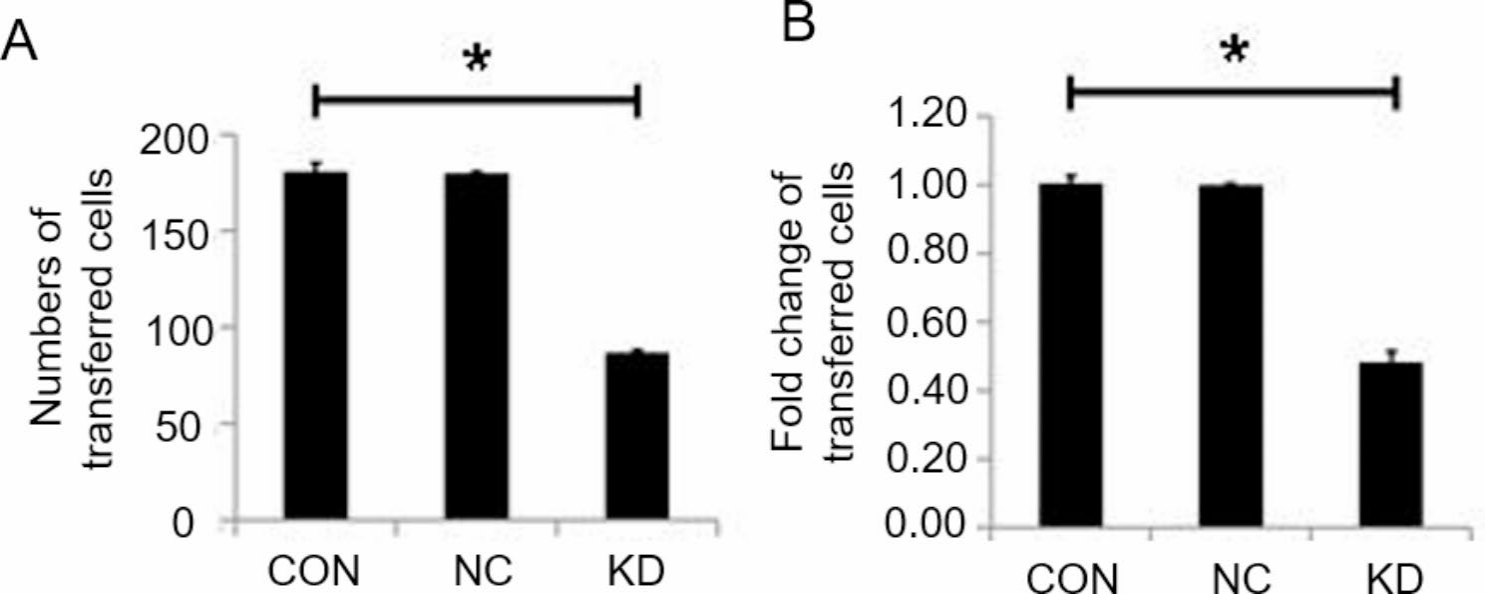
(**A**) The statistic graph of numbers of transferred cells in Transwell assay. (**B**) The statistic graph of the changes in the number of cells transferred in the transwell chamber. CON: control cells transfected with LV-CON, KD: cells transfected with Emi1-RNAi, NC: cells transfected with LV-GFP virous. All data were expressed as Mean ± SD, ^*^P < 0.05

To further study the effect of the Emi1 gene on cell migration ability, the authors compared the migration ability of cells in different transfection groups using the Transwell assay. The results showed that the cell transfer rate in the Emi1-siRNA KD group was reduced when compared with the CON group (transfer rate of < 50%) (P < 0.05) (Fig. 3A and B). The results revealed that Emi1 KD reduced the migration ability of breast cancer cells.

### Cell viability

After the transfection of the Emi1-siRNA in the KD group and the NC group, the OD value of the cells in the NC group was 0.980 ± 0.101, which was significantly higher than the OD value of the KD group (0.561 ± 0.077). The difference between the two groups was statistically significant. The results showed that Emi1 KD decreased the cell viability of breast cancer cells.

### Knockdown of Emi1 reduced the expression of proliferation-related and invasion-related genes

**Fig. 4:**
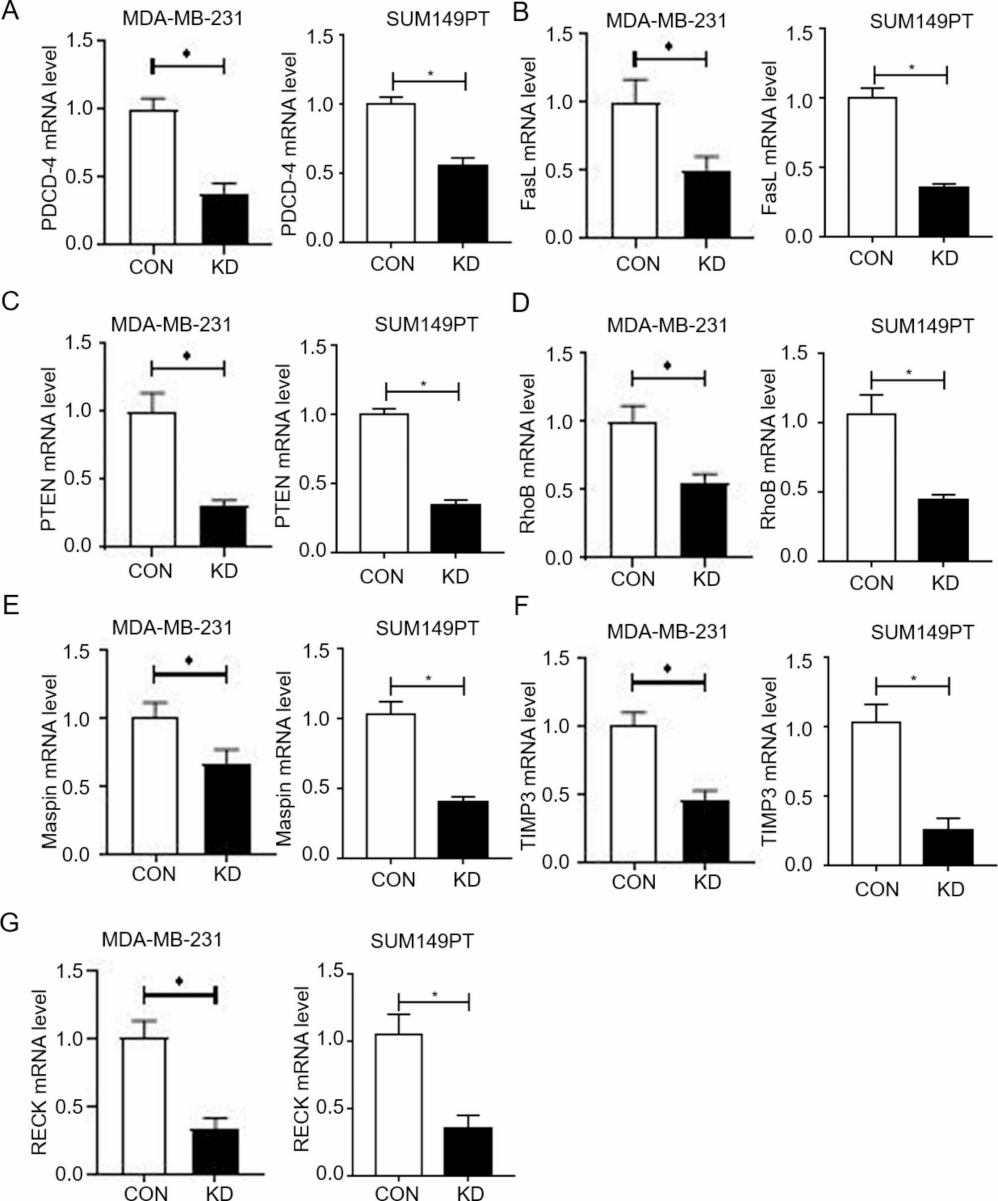
(**A**-**D**) The statistic graph of proliferation-related genes mRNA level in different groups. (**E**-**G**) The statistic graph of invasive-related genes mRNA level in different groups. CON: control cells transfected with LV-CON, KD: cells transfected with Emi1-RNAi, All data were expressed as Mean ± SD, *P < 0.05

To further study the relationship between Emi1 and cell proliferation/invasion, siRNA was used to interfere with Emi1 cellular expression. The results showed that the mRNA levels of PDCD-4, FasL, PTEN and RhoB in the KD group were significantly lower than those in the CON group after Emi1 KD (2× higher in the CON group than in the KD group) (Fig. 4A–D). The mRNA levels of Maspin, TIMP3 and RECK in the MDA-MB-231 and SUM149PT cells were significantly lower than those in the CON group after Emi1 KD by siRNA (2× higher in the CON group than in the KD group) (Fig. 4E–G). These results indicate that Emi1 is involved in the regulation of cell proliferation and invasion activities. The findings revealed that the KD of Emi1 decreased the expression of proliferation-related and invasion-related genes.

## Discussion

In this study, siRNA was used to knock down the expression of the Emi1 gene in MDA-MB-231 and SUM149PT cells, and Emi1 was found to have an important role in regulating tumour cell proliferation and migration. After constructing a lentivirus containing Emi1-siRNA, the study authors found that Emi1-siRNA could significantly reduce the expression of Emi1 in MDA-MB-231 and SUM149PT cells. Additionally, the results showed that the KD of Emi1 reduced the proliferation and migration abilities of tumour cells and downregulated the expression of proliferation-related and migration-related genes.

The Emi1 oncogene is an important cell cycle regulator that can promote the S and G2 phases by inhibiting the activity of the anaphase-promoting complex/cyclic body (APC/C); it is also related to the process of mitosis [9, 10]. Emi1 is highly expressed and plays an important role in some cancers; it is highly expressed in cancer and promotes tumour progression [11]. In chronic myeloid leukaemia (CML), Bcr-Abl enhances the phosphorylation stability of Emi1, thereby preventing the degradation of SKP2 and promoting the proliferation of CML cells [12]. Emi1 is highly expressed in breast, liver and colon cancer, and its expression is related to the degree of tumour malignancy [13].

Ribonucleic interference technology plays a very important role in the field of gene therapy for malignant tumours and has attracted much attention [14]. Moustafa et al. [13] reported that the downstream effects of Emi1 downregulation that contribute to poly-adenosine diphosphate ribose polymerase inhibitor (PARPi) resistance are increasing the concentration of the RAD51 protein in triple-negative breast cancer (TNBC) cells. They showed that the KD of Emi1 in TNBC reduced cell viability, which is consistent with the results of the present study. However, they did not investigate the effect of Emi1 KD on the invasion ability of breast cancer cells. Marzio et al. [4] also reported that cellular levels of RAD51 are kept in check by Emi1-mediated degradation. The downregulation of Emi1 in BRCA1-deficient breast cancer cells induces primary and acquired resistance to PARPi both in vitro and in vivo. The present study detected the expression of proliferation-related and invasion-related genes in breast cancer cell lines in the siRNA KD group, and all showed a significant decline, confirming that the Emi1 gene is involved in the biological process of breast cancer cells in vitro.

During the occurrence and development of breast cancer, a variety of molecules are involved in the regulation of cell proliferation and invasion, and there are Emi1 binding sites in the 3’ untranslated region of a variety of proliferation-related and invasion-related mRNAs [4]. The proteins encoded by the PDCD-4, FasL, PTEN and RhoB genes are related to the regulation of breast cancer cell proliferation, and the proteins encoded by the Maspin, TIMP3 and RECK genes are related to the regulation of breast cancer cell invasion. The PDCD-4, FasL, PTEN and RhoB genes have tumour-suppressing effects, and their expression is significantly reduced in malignant tumours, such as breast cancer and lung cancer [15, 16]. The FasL gene belongs to the superfamily of tumour necrosis factor receptors and is a signal receptor of cell apoptosis. After binding to FasL, it can induce apoptosis and activate cellular immunity to kill target cells [17]. The PDCD-4 gene is part of a class of tumour suppressors, which is located in the nucleus and can inhibit cell proliferation and promote apoptosis through the Sp transcription factor. The protein encoded by PTEN contains a loop structure with four amino acid residues inserted into the sequence, which can induce cell cycle arrest and apoptosis via the PI3K/Akt pathway [18]. Steven D. Cappell et al. [19] reported that mammalian cells commit to the cell cycle by increasing CDK2 activity and Emi1-mRNA expression to trigger a one-way APC/C^Cdh1^ inactivation switch mediated by Emi1 transitioning from a substrate to an inhibitor of APC/C^Cdh1^. After the transfection of siEmi1, the mRNA levels of proliferation-related molecules PDCD-4, FasL, PTEN and RhoB in breast cancer cell strains were significantly reduced. This confirms that Emi1 KD can regulate the proliferation-related proteins PDCD-4, FasL, PTEN and RhoB in breast cancer cells.

Maspin, TIMP3 and RECK are genes with an invasion inhibitory effect. These proteins tend to be under-expressed in breast cancer and lung cancer, which will in turn increase the invasion ability of cancer cells. Maspin belongs to the serine protease inhibitor superfamily, and it is a type of secreted protein that can block the binding of urokinase plasminogen activator (uPA) to the receptor uPA receptor and inhibit cell invasion [20]. The protein encoded by RECK contains three serine hydrolase inhibitor regions; it inhibits the secretion and activity of a variety of MMPs and can hinder the degradation of extracellular matrix by MMP2 and MMP9 [21]. Tissue inhibitors of metalloproteinases (which inhibit of the tissue pathway of metalloproteinases) can directly bind to MMPs and inhibit their functions, blocking the degradation of the extracellular matrix [22]. S Vaidyanathan et al. [23] reported that Emi1 overexpression promotes chromosome instability, and the formation of solid cancers in vivo indicates that Emi1 overexpression actively drives solid tumorigenesis. After the transfection of siEmi1 mimics, the mRNA levels of the invasion-related genes Maspin, TIMP3 and RECK in breast cancer cell strains were significantly reduced. This indicated that Emi1 KD can regulate the invasion-related genes Maspin, TIMP3 and RECK in breast cancer cells.

The lentiviral vector used in the current study has an extremely high transfection efficiency and can easily transfect the target gene into cells, which is a very effective method of gene manipulation. Based on this, the authors of the present study performed relevant tests at the tumour cell level. However, the study has several limitations: (1) the authors did not study whether the proliferation and migration of breast cancer cells in vivo are related to the expression of Emi1. In the next study, a lentivirus will be used to infect breast cancer cells in vivo and knock down the expression of Emi1. The role of Emi1 in the proliferation and metastasis of breast cancer cells will be further confirmed by monitoring the occurrence and development of tumours in mice. (2) The study results only confirmed that KD can inhibit the proliferation and metastasis of breast cancer cells. Examining the overexpression of Emi1 in tumour cells is still required for more in-depth research on whether Emi1 directly or indirectly regulates proliferation-related and metastasis-related genes. Further studies are needed to clarify the molecular mechanism by which Emi1 regulates breast cancer proliferation and invasion.

## Conclusions

In this study, siRNA was used to downregulate the high expression of the Emi1 gene in breast cancer cells, inhibit downstream signal transduction and achieve the effect of downregulating the expression of downstream target genes, thereby decreasing the proliferation and invasion ability of tumours. Additionally, it provides a cell model for subsequent in vivo experiments as well as an experimental basis for the targeted treatment of breast cancer.

### Electronic supplementary material

Below is the link to the electronic supplementary material.

Supplementary Material 1

Supplementary Material 2

Supplementary Material 3

Supplementary Material 4

Supplementary Material 5

Supplementary Material 6

Supplementary Material 7

Supplementary Material 8

Supplementary Material 9

Supplementary Material 10

Supplementary Material 11

Supplementary Material 12

## Data Availability

All data generated or analyzed during this study are included in this published article.
