# Supplementary figures and images for: Effect of Emi1 gene silencing on the proliferation and invasion of human breast cancer cells
Source: BMC Mol Cell Biol. 2023 Dec 1;24:34. doi: 10.1186/s12860-023-00494-1 (PMC10690968; doi:10.1186/s12860-023-00494-1)

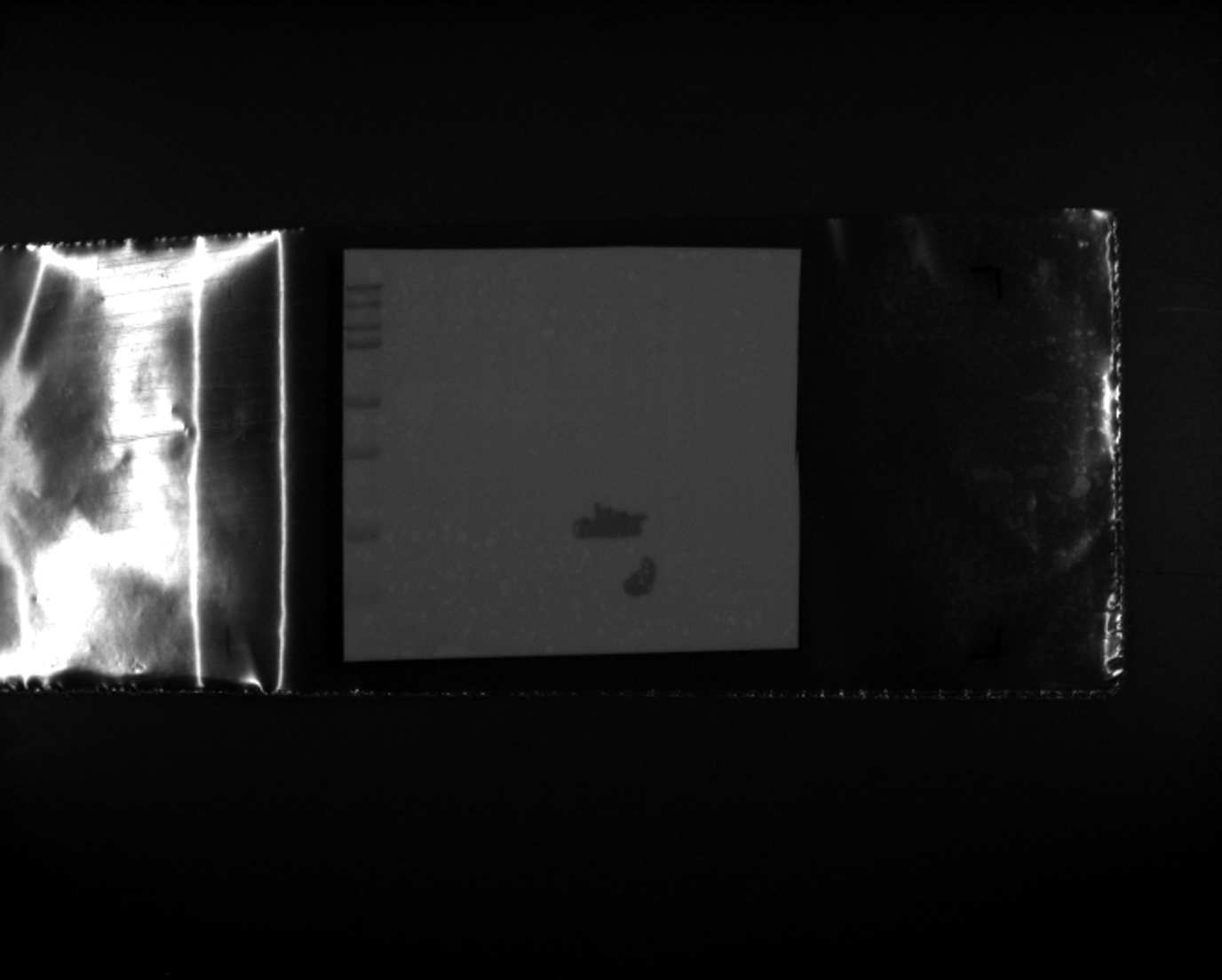

Supplement: Supplementary file 1 — Supplementary Material 1 [file 12860_2023_494_MOESM1_ESM.png]

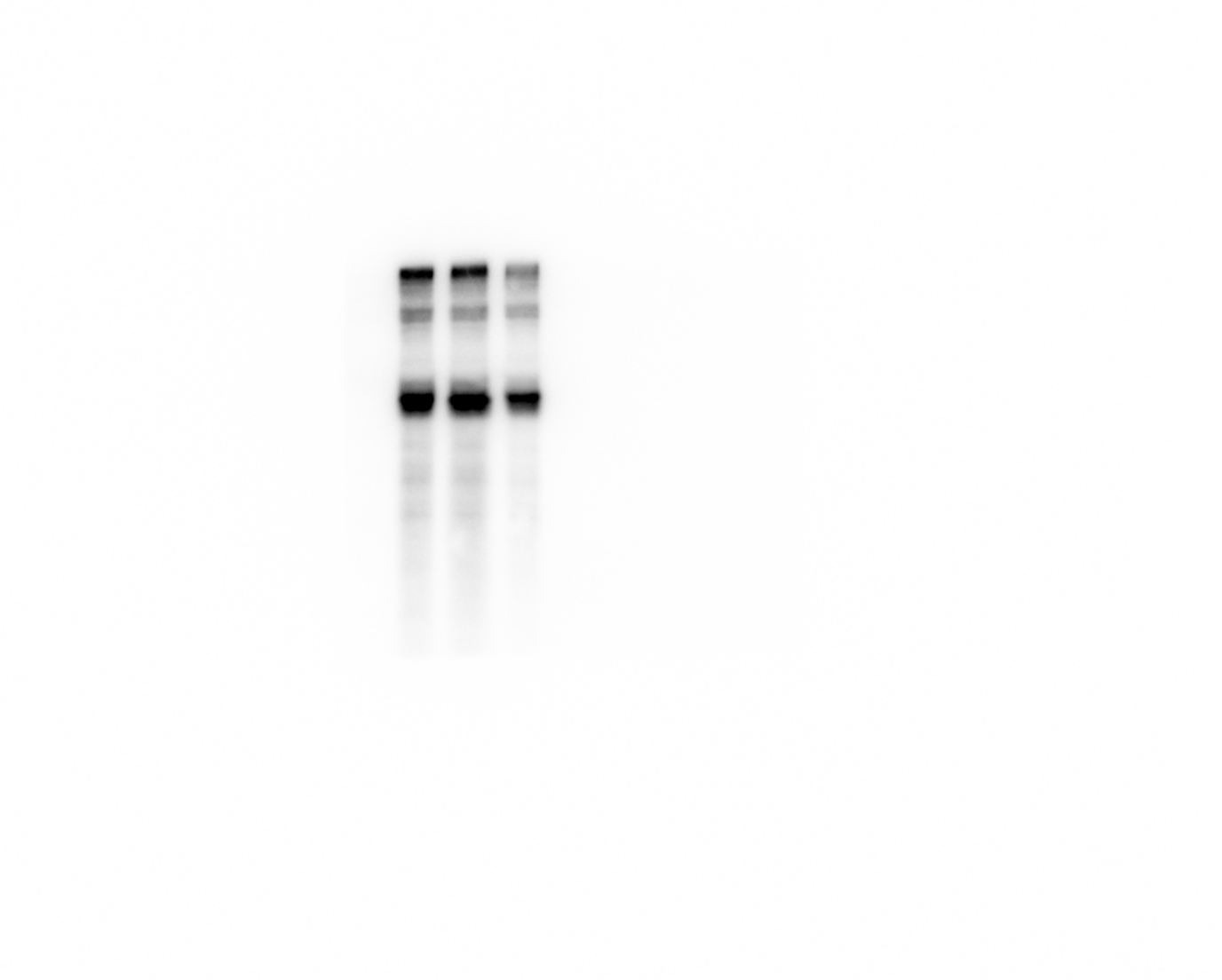

Supplement: Supplementary file 2 — Supplementary Material 2 [file 12860_2023_494_MOESM2_ESM.png]

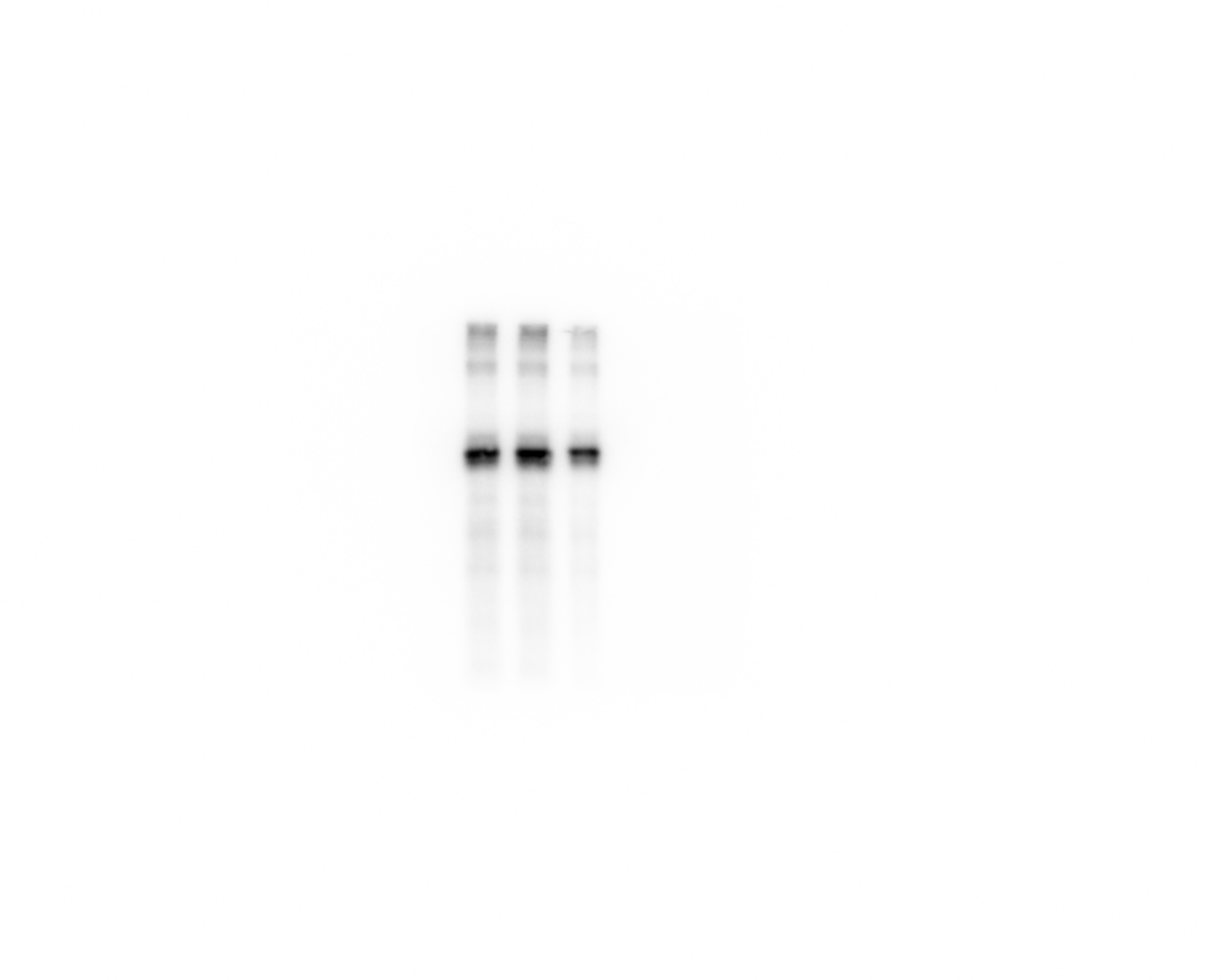

Supplement: Supplementary file 3 — Supplementary Material 3 [file 12860_2023_494_MOESM3_ESM.png]

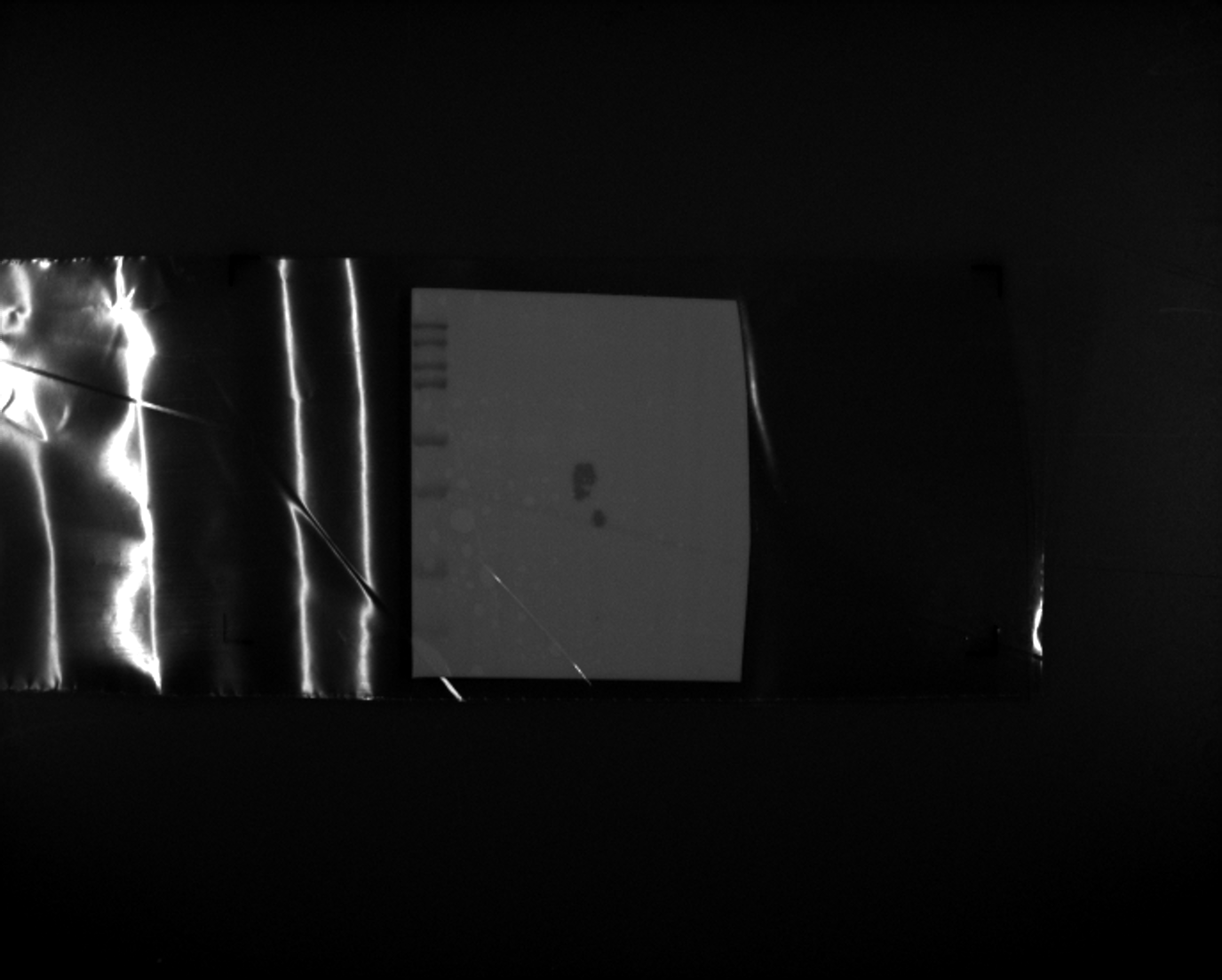

Supplement: Supplementary file 4 — Supplementary Material 4 [file 12860_2023_494_MOESM4_ESM.png]

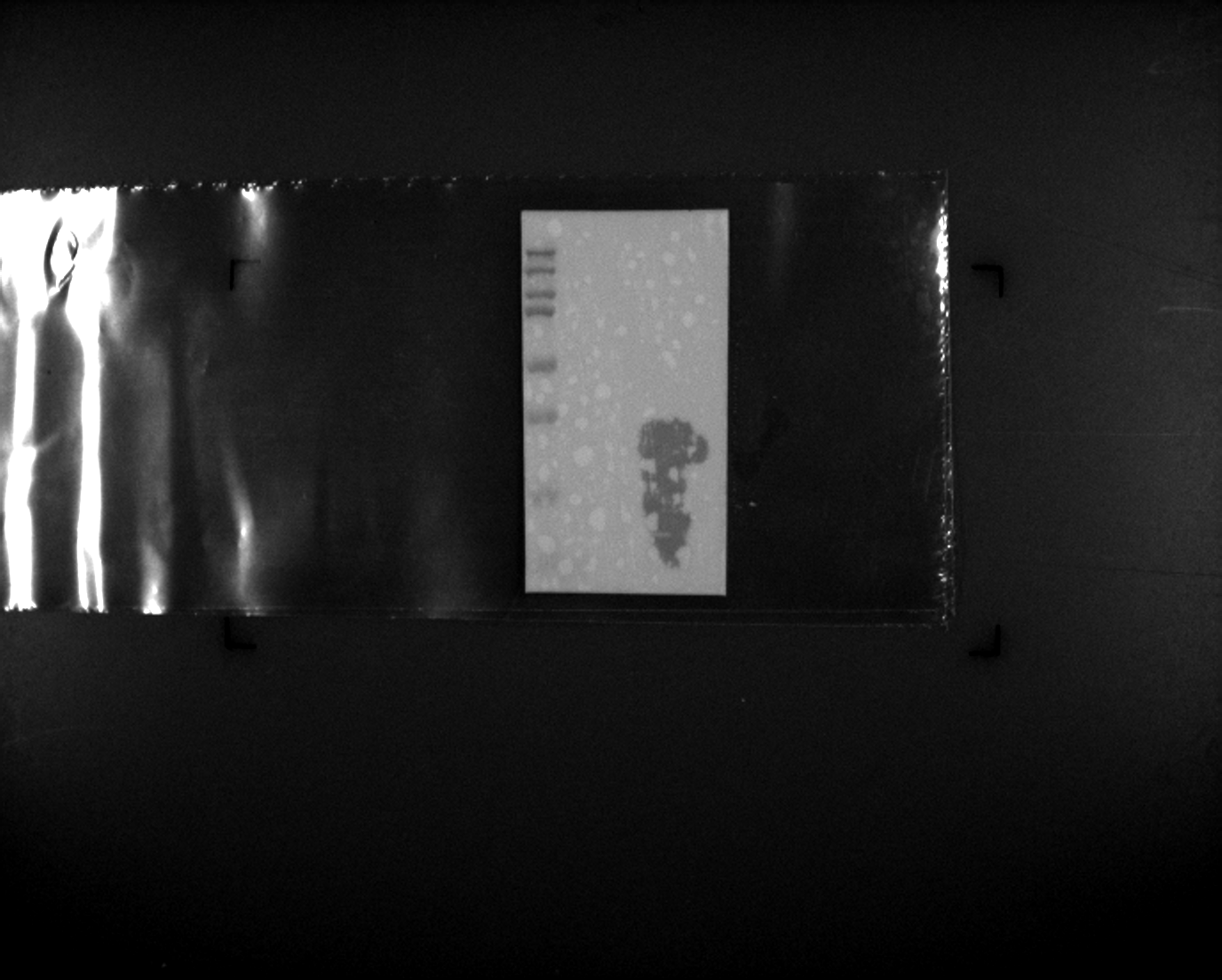

Supplement: Supplementary file 5 — Supplementary Material 5 [file 12860_2023_494_MOESM5_ESM.png]

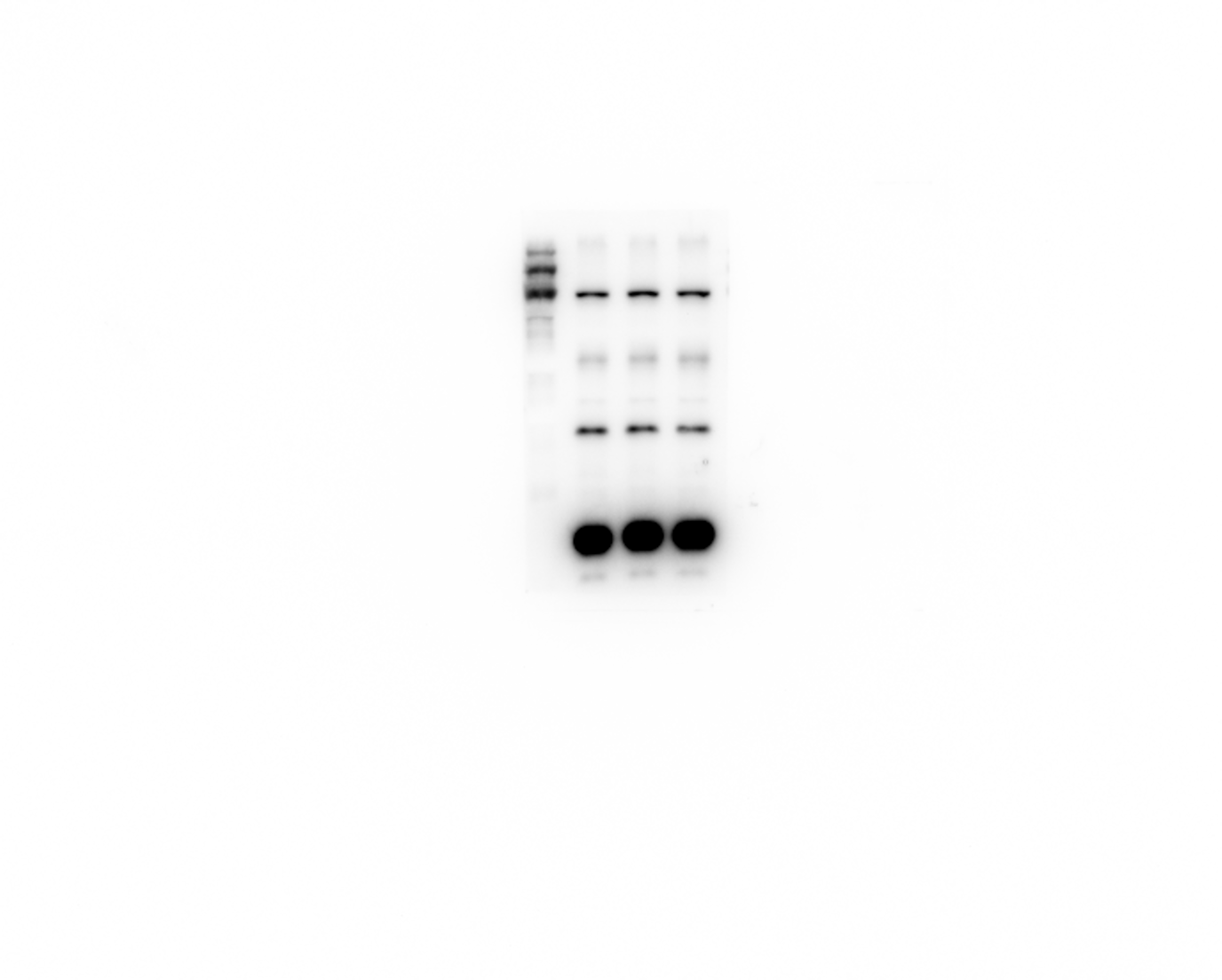

Supplement: Supplementary file 6 — Supplementary Material 6 [file 12860_2023_494_MOESM6_ESM.png]

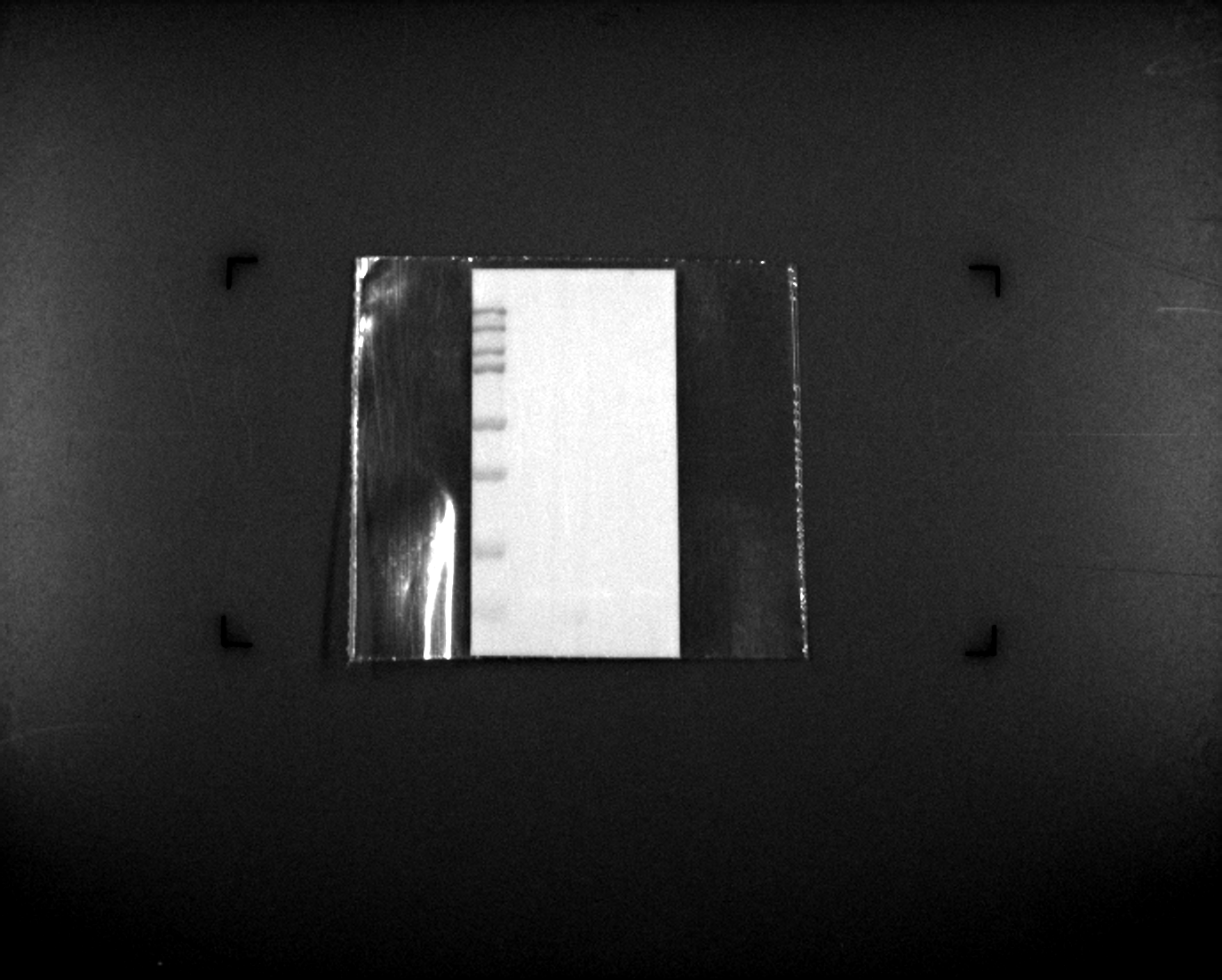

Supplement: Supplementary file 7 — Supplementary Material 7 [file 12860_2023_494_MOESM7_ESM.png]

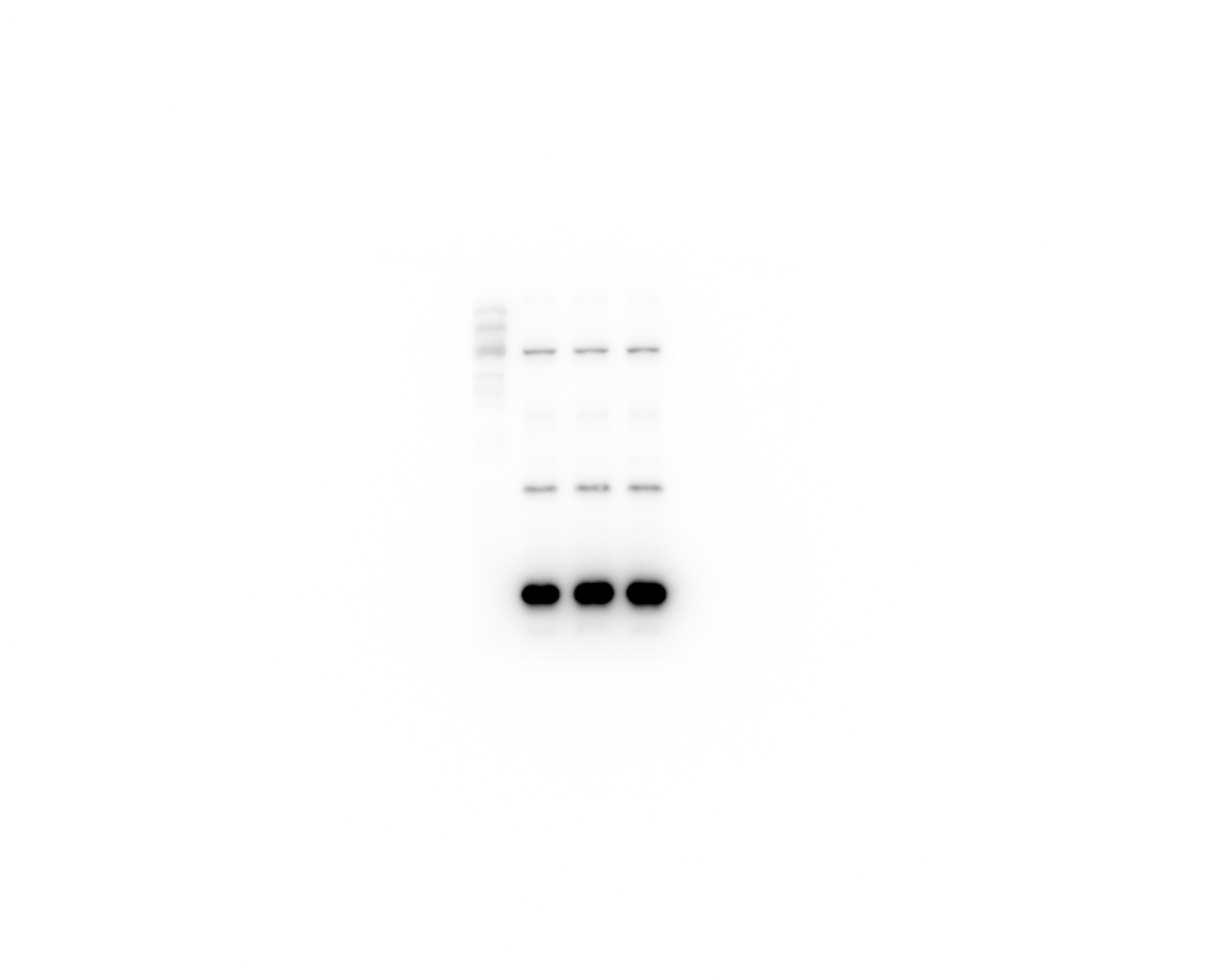

Supplement: Supplementary file 8 — Supplementary Material 8 [file 12860_2023_494_MOESM8_ESM.png]

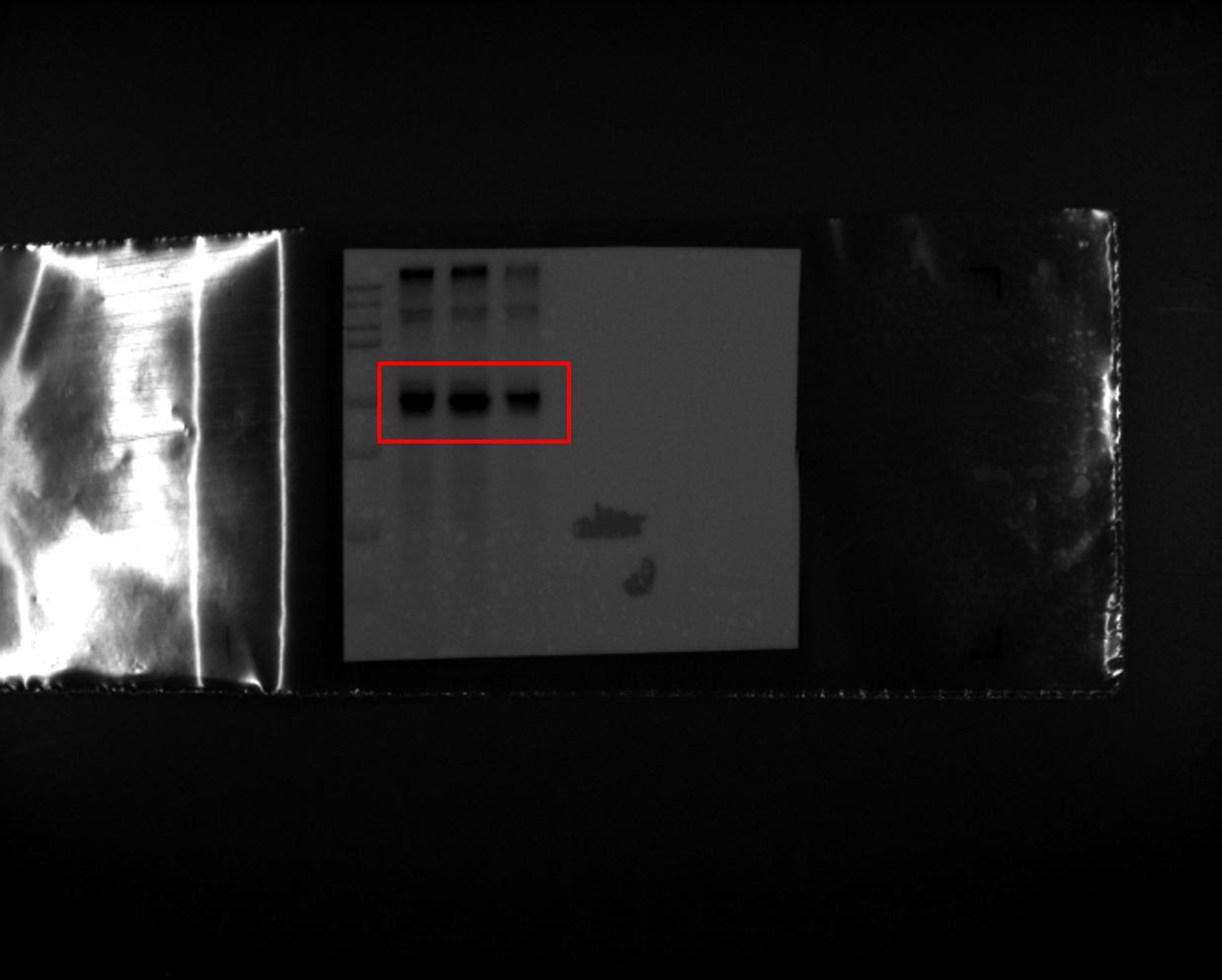

Supplement: Supplementary file 9 — Supplementary Material 9 [file 12860_2023_494_MOESM9_ESM.png]

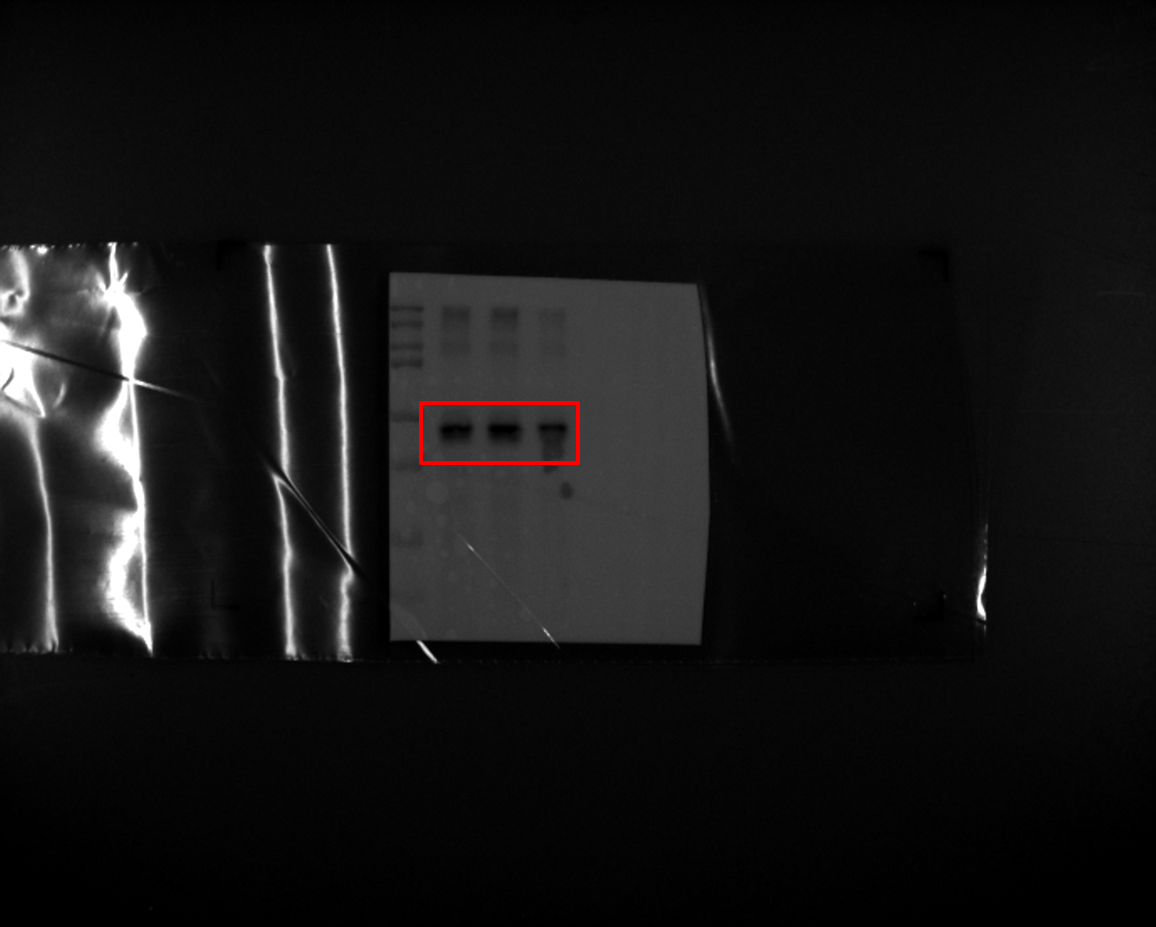

Supplement: Supplementary file 10 — Supplementary Material 10 [file 12860_2023_494_MOESM10_ESM.png]

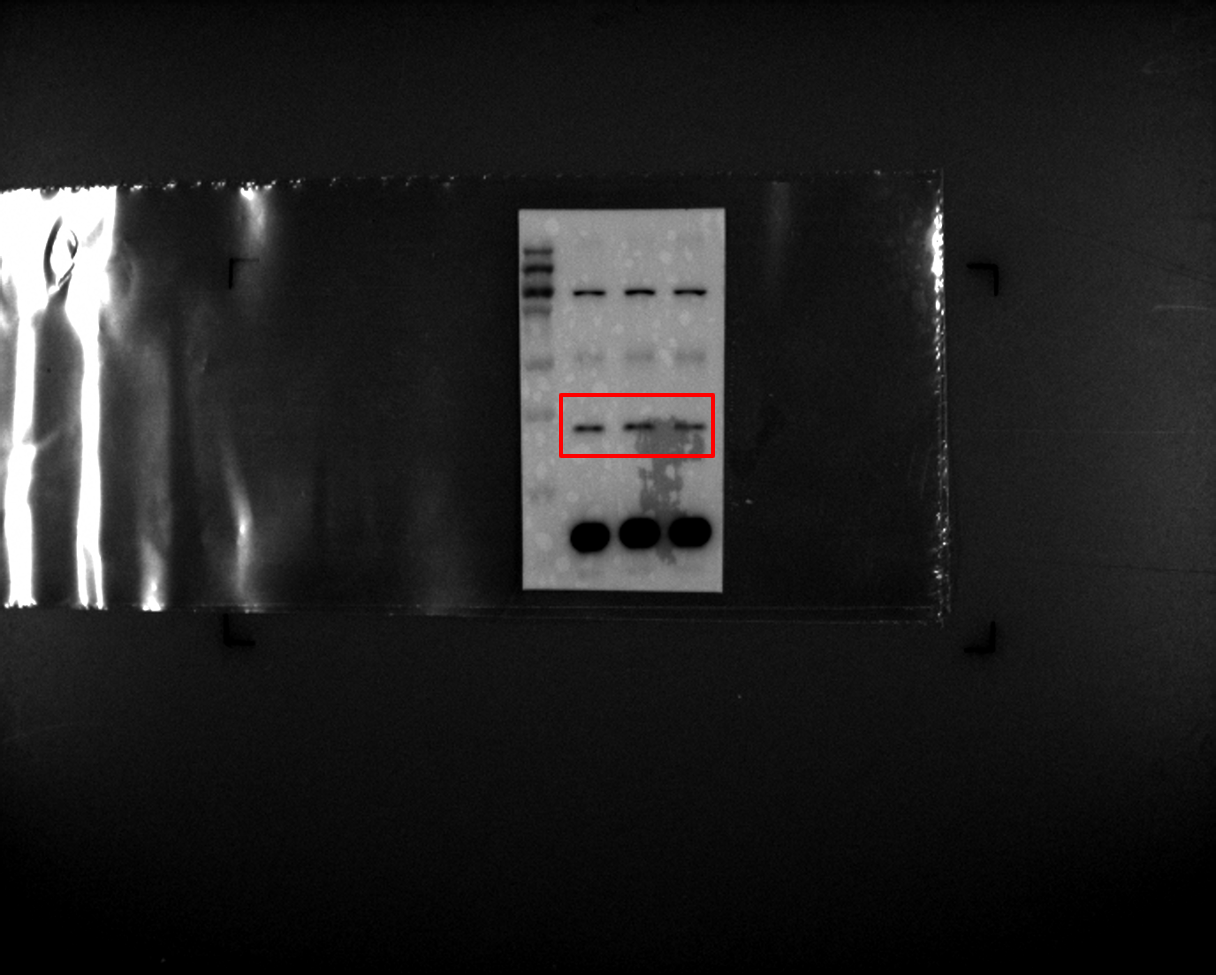

Supplement: Supplementary file 11 — Supplementary Material 11 [file 12860_2023_494_MOESM11_ESM.png]

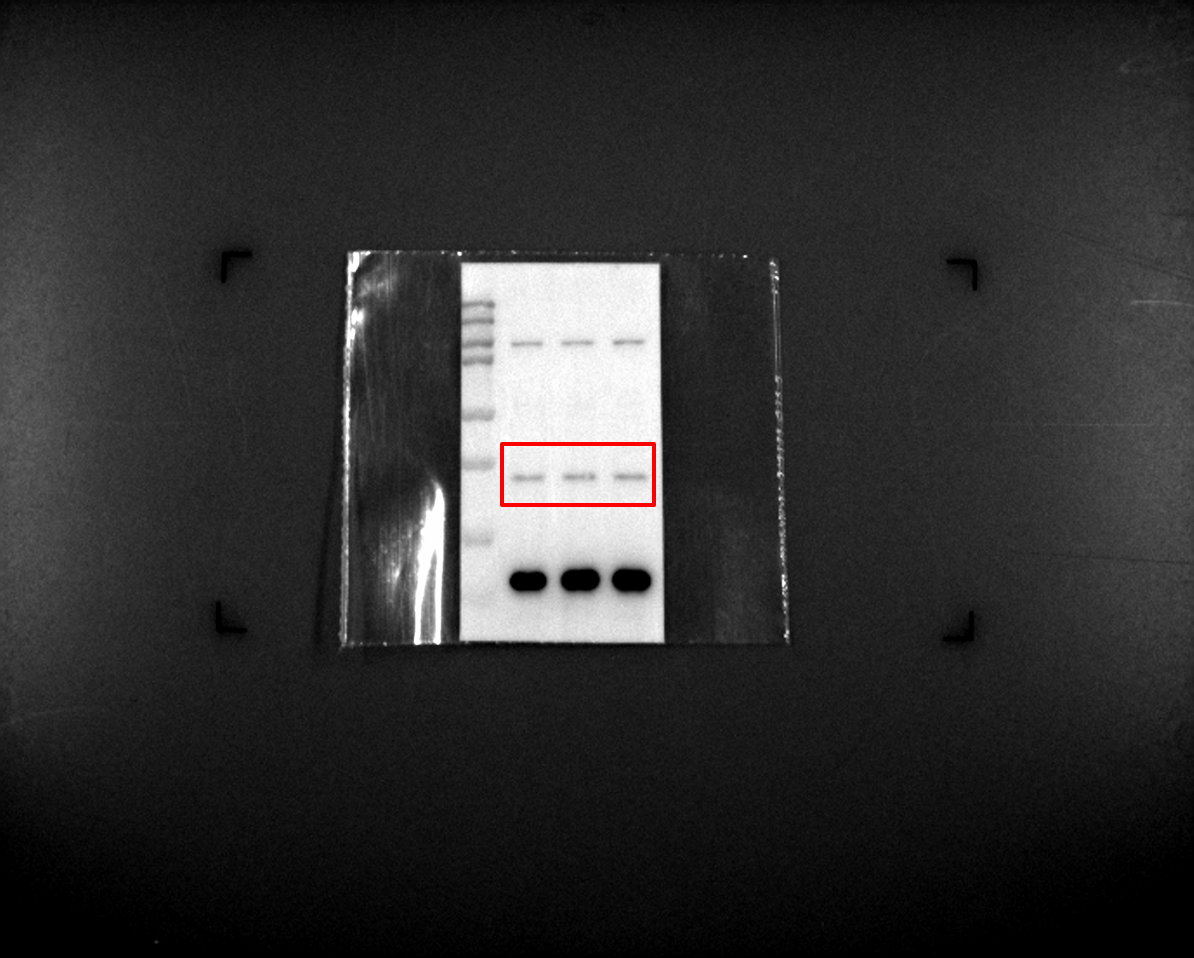

Supplement: Supplementary file 12 — Supplementary Material 12 [file 12860_2023_494_MOESM12_ESM.png]
